# Impact of menopause on clinical periodontal outcomes: a systematic review

**DOI:** 10.1007/s00784-026-06813-y

**Published:** 2026-03-23

**Authors:** Fabiola Civiletto-S. Martín, Maria J. Rus, Angela de la Cruz Gándara Alvarez, Aurea Simon-Soro, Cristiane Cantiga-Silva

**Affiliations:** Departamento de Estomatología, Facultad de Odontología, Universidad de Sevilla, Seville, Spain

**Keywords:** Menopause, Periodontal health, Estrogen deficiency, Postmenopausal women, Oral inflammation

## Abstract

**Objectives:**

To investigate whether menopause is associated with adverse clinical periodontal outcomes by systematically comparing postmenopausal and premenopausal women.

**Materials and methods:**

A systematic review of observational studies was conducted following PRISMA guidelines. Electronic databases were searched up to March 2025 March 2025 for observational studies reporting clinical periodontal parameters in postmenopausal women not undergoing hormone replacement therapy. Risks of bias and certainty of evidence were assessed using ROBINS-I and GRADE frameworks.

**Results:**

Nine studies met inclusion criteria. Postmenopausal women showed greater clinical attachment loss, increased probing depths, and more pronounced signs of inflammation. However, the certainty of evidence was rated as moderate to low, mainly due to methodological variability.

**Conclusions:**

While the observed trends suggest that menopause is a clinically relevant factor in periodontal health, the current evidence base is weak. High-quality prospective studies are urgently needed to confirm these findings before informing clinical practice or developing new guidelines.

**Clinical relevance:**

Findings contribute to the current understanding of systemic influences on periodontal disease, with relevance to aging, women’s health, and menopause as a physiological stage of life.

**Graphical abstract:**

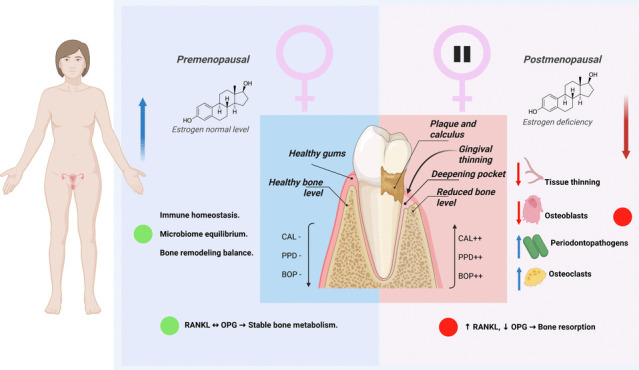

**Supplementary Information:**

The online version contains supplementary material available at 10.1007/s00784-026-06813-y.

## Introduction

Periodontitis is a multifactorial and chronic inflammatory disease that leads to the gradual destruction of the periodontal ligament and alveolar bone. Although the presence of bacterial biofilm is recognized as the principal initiating factor, the immune response of the individual and various systemic modifiers plays a crucial role in determining the severity and progression of the disease [1]. Among these systemic factors hormonal fluctuations related to menopause have emerged as a particularly relevant factor, drawing increasing attention due to their potential impact on periodontal health.

Menopause is associated with a marked decrease in circulating estrogen levels, which has been shown to affect bone remodeling and immune modulation [2]. Estrogen receptors are expressed in periodontal tissues, including gingival fibroblasts and periodontal ligament cells, suggesting that estrogen may play a direct role in maintaining periodontal homeostasis [3]. Estrogen deficiency may promote an exaggerated inflammatory response, favoring increased expression of pro-inflammatory cytokines such as TNF-α and IL-6, which contribute to periodontal tissue breakdown and bone loss [4, 5].

Beyond immune modulation, hypoestrogenism may also influence the subgingival microbial ecosystem. Recent findings indicate that postmenopausal women exhibit a distinct microbial profile compared to premenopausal women, with an increased abundance of periodontopathogens [6, 7]. This microbial shift is accompanied by local inflammatory changes, as reflected in altered levels of salivary and gingival crevicular fluid (GCF) biomarkers. Notably, reductions in protective molecules such as osteoprotegerin (OPG) and increases in inflammatory mediators have been documented [3, 5], suggesting that both host and microbial factors converge to shape periodontal vulnerability in menopause.

Although several studies have explored the relationship between menopause and periodontal health, the evidence remains inconclusive. While some report increased clinical attachment loss in postmenopausal women [7], others find no significant differences when compared to premenopausal counterparts [4]. This variability may be explained by differences in study design, time since menopause, use of hormone replacement therapy (HRT), and the presence of confounding factors such as age or systemic conditions [8, 9]. Consequently, the extent to which estrogen deficiency influences periodontal health remains uncertain.

To address this gap, we conducted a systematic review aimed at evaluating clinical periodontal outcomes in postmenopausal versus premenopausal women, with particular emphasis on identifying potential associations with periodontal disease progression or severity.

## Materials and methods

### Protocol registration and reporting format

This systematic review was conducted in accordance with the Preferred Reporting Items for Systematic Reviews and Meta-Analyses (PRISMA) statement [10] and Cochrane guidelines for systematic reviews, version 6.5 [11]. The review protocol was prospectively registered in the International Prospective Register of Systematic Reviews (PROSPERO) under the registration CRD420251055123.

### Review question

In postmenopausal women compared to premenopausal women, are there clinical changes such as periodontal attachment loss, alveolar bone resorption, and worsening of periodontal disease?

### PICO question (Problem, Intervention, Comparison, Outcome)

Population (P): Postmenopausal women and no history of HRT.

Intervention (I): Postmenopausal state.

Comparison: (C): Premenopausal women.

Outcomes (O): Worsening of periodontitis and periodontal health parameters, including clinical attachment loss (CAL), probing pocket depth (PPD), gingival recession (GR), bleeding on probing (BOP), gingival index (GI), plaque index (PI).

### Eligibility criteria

Inclusion criteria were established as (a) Studies including premenopausal and postmenopausal women group; (b) human studies; (c) studies analyzing the effects of estrogen deficiency on periodontal tissues; (d) studies published in English or Spanish.

The exclusion criteria were as follows: (a) studies that do not provide a differentiated analysis for postmenopausal women; (b) studies that do not specifically analyze the effects of estrogen deficiency on periodontal tissues; (c) animal studies, *in vitro* studies, narrative reviews, opinion articles, and single case reports; (d) studies published in languages other than English or Spanish; (e) studies that do not meet the inclusion criteria.

### Information sources and searches

A comprehensive literature search was conducted using the electronic databases PubMed, Embase, Scopus and Web of Science to identify relevant studies. The initial search strategy was developed in PubMed using a combination of Medical Subject Headings (MeSH) and free-text terms. It was subsequently refined with contributions from the research team (AS-S, CC-S, MJR), whose expertise in oral health and menopause ensured comprehensive coverage of the core concepts: menopause, estrogen deficiency, and periodontal diseases. The strategies were adapted to the controlled vocabulary and syntax specific to each database. No restrictions were applied regarding the date of publication. Only studies published in English or Spanish were included. The final search was completed on 23 March 2025.

The detailed strategies used were as follows:

- PubMed: ("Postmenopause"[MeSH] OR postmenopausal women OR menopause) AND (estrogen* OR "Estrogen Deficiency"[MeSH] OR hypoestrogenism OR "estrogen depletion" OR "estrogen loss" OR "low estrogen levels") AND ("Periodontal Diseases"[MeSH] OR periodontitis OR "gingival recession" OR "alveolar bone loss" OR "gingival thinning" OR "periodontal tissue change*").
- EMBASE: ('postmenopausal woman'/exp OR "postmenopausal women" OR postmenopausal OR menopause) AND (estrogen* OR 'estrogen deficiency'/exp OR hypoestrogenism OR "estrogen depletion" OR "estrogen loss" OR "low estrogen levels") AND ('periodontal disease'/exp OR periodontitis OR 'gingival recession'/exp OR 'alveolar bone loss'/exp OR "gingival thinning" OR "periodontal tissue change*").
- SCOPUS: TITLE-ABS-KEY ((postmenopausal OR "postmenopausal women" OR menopause) AND estrogen* OR "estrogen deficiency" OR hypoestrogenism OR "estrogen depletion" OR "low estrogen levels") AND ("periodontal disease*" OR periodontitis OR "gingival recession" OR "alveolar bone loss" OR "gingival thinning" OR "periodontal tissue change*")).
- Web of Science: ("postmenopausal OR "postmenopausal women" OR menopause) AND (estrogen* OR "estrogen deficiency" OR hypoestrogenism OR "estrogen depletion" OR "estrogen loss" OR "low estrogen levels") AND ("periodontal disease*" OR periodontitis OR "gingival recession" OR "alveolar bone loss" OR "gingival thinning" OR "periodontal tissue change*")

All search results were imported into reference management software, and duplicates were removed prior to screening. Grey literature, clinical trial registries, or conference proceedings were not included in the search strategy.

### Screening process and data extraction

Duplicates were removed and screening of titles and abstracts was performed independently by two reviewers (FC-SM, CC-S) using Rayyan QCRI to facilitate the blinded selection process and conflict resolution [12]. Full texts of potentially eligible studies were then assessed for inclusion based on predefined criteria. Disagreements were resolved by consensus or consultation with a third reviewer (AS-S). Reasons for exclusion were recorded, and inter-reviewer agreement was measured using the Kappa coefficient.

Data extraction included the author, year of publication, study design, sample size, characteristics of participants, estrogen level or status confirmation, periodontal parameters evaluated (PPD, CAL, BOP, PI, GI, GR), presence of systemic diseases, smoking status, and key statistical outcomes. In addition, the presence or absence of HRT was recorded. The outcome variables were categorized into clinical domains according to predefined PICO criteria. Only studies that clearly compared premenopausal and postmenopausal women without HRT were included.

### Quality of evidence assessment

Risk of bias was independently assessed by two reviewers (CC-S, MJR) using a structured approach based on the ROBINS-I (Risk of Bias in Non-randomized Studies – Interventions, version 2) tool, adapted for cross-sectional observational designs. Although originally intended for evaluating intervention studies, the framework was applied to assess methodological limitations relevant to cross-sectional studies in the present review. The assessment considered seven domains: (D1) bias due to confounding; (D2) bias in selection of participants; (D3) bias in classification of exposures; (D4) bias due to deviations from intended exposures; (D5) bias due to missing data; (D6) bias in measurement of outcomes; and (D7) bias in selection of the reported result. Each domain was rated as low, moderate, serious, or critical risk of bias, or as having insufficient information to permit a judgment. An overall risk of bias judgment was assigned to each study, reflecting the highest level of bias identified across the domains. Disagreements between reviewers were resolved through discussion or consultation with a third reviewer (AS-S). The certainty of the evidence and strength of recommendations were intended to be evaluated using the GRADE (Grading of Recommendations Assessment, Development and Evaluation) approach, contingent upon data availability and consistency across studies [13]. A quantitative meta-analysis was not conducted due to significant heterogeneity in study designs, populations, and outcome measurements.

## Results

### Search results

**Fig. 1:**
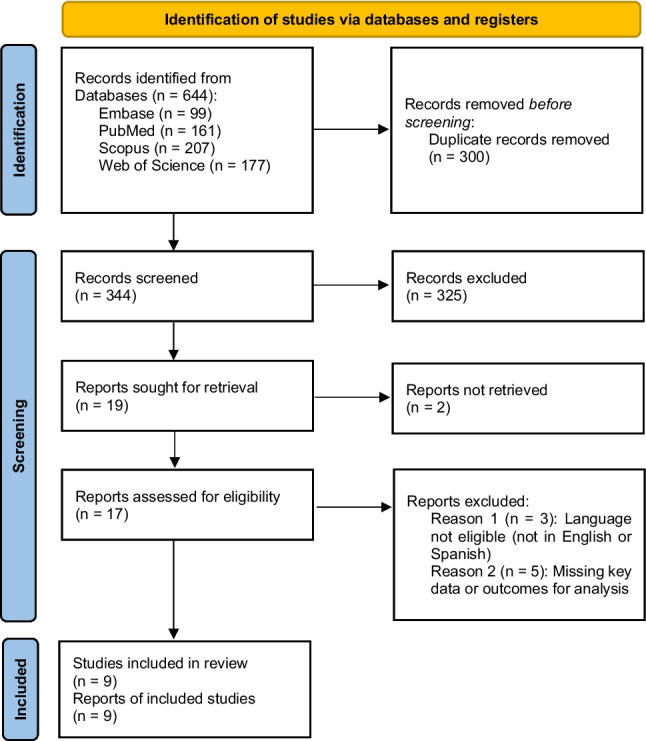
Study selection process following PRISMA 2020. Flow diagram summarising the identification, screening, and inclusion of eligible observational studies assessing the association between menopause and periodontal health. Of 644 records initially retrieved, 9 studies were included in the final qualitative synthesis after applying inclusion and exclusion criteria

A total of 644 records were identified through database searches (Fig. 1). After removal of duplicates (*n* = 300), 344 articles remained for title and abstract screening. Based on predefined eligibility criteria, 19 articles were selected for full-text assessment. Nine studies were included in the qualitative synthesis (Table 1). Inter-rater agreement for study selection indicated a high level of consistency between reviewers (κ = 0.92), with any discrepancies resolved through discussion to reach consensus.

**Table 1:** Summary of included studies on menopause and periodontal parameters

Authors/ Year		Study design	Sample size		Confirmation method		Variables		Systemic diseases		Smoking		Key finding	Statistical results
Yakar et al. 2025 [7]		Cross-sectional	229 women (116 pre; 113 post)		Serum estradiol mesures		Periodontal Status; PPD, BOP, PI,		38% pre; 58% post		32% pre; 26% post		Increased periodontitis stage 3&4 in postmenopausal women	PPD: 2.2 (2.0–2.7) pre/ 2.4 (2.1–2.9) post (*p* = 0.037); BOP and PI not significant; Stage 3 & 4 periodontitis: 13 (11%) pre/ 24 (21%) post (*p* = 0.039)
Yakar et al. 2024 [5]		Cross-sectional	146 women (71 pre; 75 post)		Menopause clinically confirmed		Periodontal Status; Salivary cytokines (TNF-α, IL-6, IL-10, IL-7), PPD, BOP		Excluded		29% pre; 34% post		Salivary cytokines levels are higher in premenopausal women	PPD and BOP not significant; Salivary cytokines (TNF-α: 307 (178–362) pre/ 153 (105–288) post (*p* < 0.000); IL-10: 556 (406–668) pre/ 363 (145–650) post (*p* = 0.002); IL-7: 43 (17–64) pre/ 19 (10–45) post (*p* = 0.021); IL-6: not significant
Agrawal et al. 2021 [15]		Cross-sectional	60 women (30 pre; 30 post)		Menopause clinically confirmed		Periodontal Status; PPD, CAL, PI, GI		Excluded		Non-smokers		Periodontitis are more common in postmenopausal women	(Mean ± SD) PPD: 1.56 ± 0.44 mm pre/ 4.36 ± 1.34 mm post (*p* = 0.01); CAL: 1.57 ± 0.45 mm pre/ 5.08 ± 1.45 mm post (*p* = 0.01); PI: 0.96 ± 0.36 mm pre/ 1.94 ± 0.46 mm post (*p* = 0.01); GI: 0.98 ± 0.48 mm pre/ 1.78 ± 0.44 mm post (*p* = 0.01)
Agrawal et al. 2018 [4]		Cross-sectional	80 women (40 pre; 40 post)—comparison between PD groups (*n* = 40)		Menopause clinically confirmed		Salivary cytokines (TNF-α), PPD, CAL, GBI, GI		Excluded		Non-smokers		Postmenopause increases TNF-α without clinical worsening of periodontitis	PPD, CAL, GBI and GI: not significant; Salivary cytokine TNF-α: 4.25 ± 1.20 pre/ 5.13 ± 1.35 post (*p* = 0.041)
Thomas 2014 [6]		Cross-sectional	220 women (110 pre; 110 post) comparison between PD groups (*n* = 220)		Not reported		Chronic periodontitis, Mild periodontitis/ Gingivitis		Excluded		Not reported		Chronic periodontitis are common in postmenopausal women	Chronic periodontitis: 46 (41.8%) pre/ 88 (80%) post; Mild periodontits or gingivitis: 64 (58%) pre/ 22 (20%) post
Scardina and Messina 2012 [16]		Cross-sectional	54 women (27 pre; 27 post)		Not reported		Periodontal Status; Capillary density in gingival mucosa		Excluded		Non-smokers		Lower gingival capillary density in postmenopausal women	Total Diameter in labial mucosa: (Mean ± SD) 89.62 ± 17.83 pre/ 28.86 ± 10.92 post (*p* < 0.05)
Babür et al. 2012 [3]		Prospective observational study	44 women (22 pre; 22 post)—comparison between PD groups (n = 22)		Menopause clinically confirmed		OPG levels in GCF, PI, GI, BOP, PPD, CAL		Excluded		Non-smokers		No clinical or OPG differences in periodontal disease between pre and pos woman	OPG levels in GCF, PI, GI, BOP, PPD, CAL: not significance
Zhang et al. 2010 [8]		Cross-sectional	205 women (80 pre; 125 post)		Not reported		PPD, CAL		Excluded		Not reported		Periodontitis in pre and post	(mean ± SD) PPD: 4.80 mm ± 0.70 Pre/ 4.60 mm ± 1.02 post; CAL
PD													women show similar PPD and CAL values	5.20 mm ± 0.80 pre/ 5.50 mm ± 1.00 post
Haas et al. 2009 [14]	Cross-sectional			292 women (108 pre; 184 post HRT- and HRT +)		Menopause clinically confirmed		Periodontal Status; CAL		Excluded		30.6% pre; 24.6% post HRT + ; 25.2% post HRT-	Periodontitis are more prevalence in postmenopausal women	CAL (mean): 2.22 mm pre/ 2.86 mm post HRT- (*p* = 0.003); Periodontitis: 64.4% pre/ 46.3% post HRT- (*p* = 0.005)

*PPD* Probing Pocket Depth; *CAL* Clinical Attachment Level; *BOP* Bleeding on Probing; *PI* Plaque Index; *GI* Gingival Index; *GBI* Gingival Bleeding Index; *OPG* Osteoprotegerin; *GCF* Gingival Crevicular Fluid; *HRT* Hormone Replacement Therapy; *SD* Standard Deviation; *p* *P* value

### Description of included studies

The review includes nine studies: eight cross-sectional and one prospective (Table 1). The review includes nine observational studies: eight cross-sectional and one prospective. Published between 2009 and 2024, the studies involved a total of 1,330 women, with sample sizes ranging from 44 to 292. Premenopausal participants had mean ages between 27.8 and 47.0 years, while postmenopausal women ranged from 50.0 to 57.3 years.

Confirmation of menopausal status varied. Some studies used serum estradiol levels, others relied on clinical criteria, and a few did not report the method. Yakar et al. [5] and Yakar et al. [7] grouped participants by menopausal status before assessing periodontal health. Haas et al. [14] and Agrawal et al. [15] included women with mixed periodontal status across both groups. Haas et al. [14] also analyzed the effect of HRT on periodontal attachment loss (PAL). Other studies focused only on women diagnosed with periodontitis, such as Babür et al. [3], Agrawal et al. [4], Thomas [6] and Zhang et al. [8]. Scardina and Messina [16] studied only healthy women to assess gingival microcirculation.

Most studies excluded women with systemic diseases and smokers, but reporting was inconsistent. Yakar et al. [7] and Haas et al. [14], for example, included smokers in both groups and presented comparative data. Periodontal parameters varied across studies. Investigators assessed PPD, CAL, BOP, PI, GI, GR, and periodontal diagnosis or staging. Some studies also measured salivary or crevicular biomarkers, including TNF-α, IL-6, IL-7, IL-10 and OPG, or evaluated gingival microvascular density [3–5, 16].

Several studies found worse periodontal conditions in postmenopausal women. Agrawal et al. [15] reported significantly higher mean PPD and CAL (*p* = 0.01), and Yakar et al. [7] found a higher prevalence of stage 3–4 periodontitis in the postmenopausal group (21%) compared to premenopausal (11%) (*p* = 0.039). In contrast, Babür et al. [3] and Zhang et al. [8] found no significant differences when comparing women with periodontitis. Cytokine findings also varied. Agrawal et al. [4] and Yakar et al. [5] detected higher levels of TNF-α, IL-10, and IL-7 in premenopausal women, despite similar clinical status. Haas et al. [14] showed that postmenopausal women without HRT had worse PAL and more periodontitis compared to premenopausal women.

### Quality of evidence assessment

**Fig. 2:**
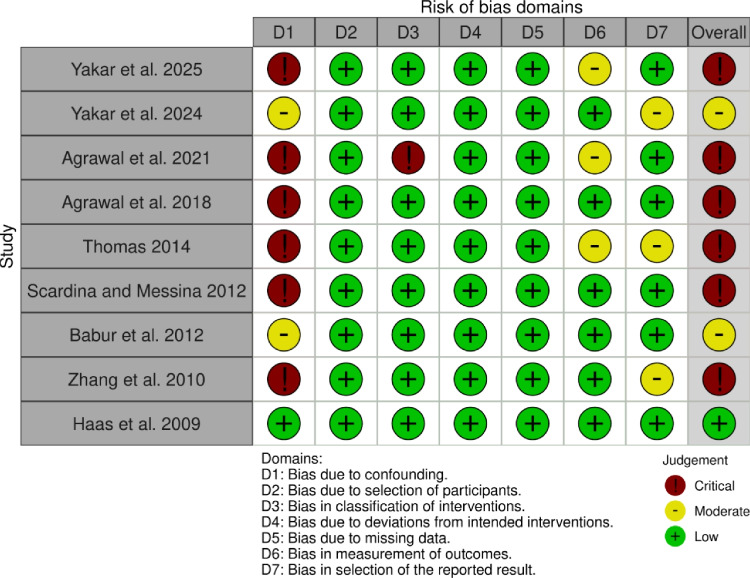
Risk of bias across included studies using the ROBINS-I tool. Risk of bias assessment for the nine observational studies included in the review, evaluated across seven ROBINS-I domains. Most studies showed moderate to critical risk, particularly due to confounding and lack of adjustment for key variables. One study was rated as low risk overall

The initial inter-rater agreement was moderate (κ = 0.55), with discrepancies in two studies [8, 15]. These were resolved through discussion and adjudication by a third reviewer (AS-S), and consensus was reached for all studies. In Fig. 2, most included studies exhibited a critical or moderate risk of bias within the confounding domain (D1), with six studies failing to adequately adjust for age or other key confounders [4, 6–8, 15, 16]. In contrast, the exposure classification (D2) and participant selection (D3) domains predominantly demonstrated low risk of bias. Regarding outcome measurement (D6), three studies showed moderate risk due to lack of examiner calibration or ambiguous outcome definitions [6, 7, 15]. For selective reporting (D7), a moderate risk of bias was identified in studies featuring multiple comparisons or insufficient transparency in analytical methods [5, 6, 8]. Only one study was assessed as having an overall low risk of bias [14].

According to the GRADE assessment (Table 2), the certainty of the evidence ranged from low to moderate. The outcome severity of periodontitis was rated as low due to risk of bias, inconsistency, and imprecision. In contrast, overall periodontal health status showed moderate certainty, supported by more consistent findings and adequate sample sizes.

**Table 2:** GRADE evidence profile: clinical impact of menopause on periodontal health

Question: In postmenopausal women compared to premenopausal women, are there clinical changes such as periodontal attachment loss, alveolar bone resorption, and worsening of periodontal disease?													
Certainty assessment													
									Impact		Certainty		Importance
№ of studies	Study design		Risk of bias	Inconsistency	Indirectness		Imprecision	Other considerations					
Severity of periodontitis													
4	non-randomised studies		seriousa	very seriousb	not serious		seriousc	all plausible residual confounding would reduce the demonstrated effect	Conflicting results. Two studies showed increased severity in postmenopausal women, while two studies found no significant difference	⨁◯◯◯ **Very lowa,b,c**			**CRITICAL**
Overall periodontal health status													
5	non-randomised studies	seriousd		seriouse	not serious	not seriousf		all plausible residual confounding would reduce the demonstrated effect	Most studies suggest a worse periodontal status in postmenopausal women, especially regarding PPD and CAL	⨁⨁⨁◯ **Moderated,e,f**		**IMPORTANT**	

a. Insufficient adjustment for confounders in several studies.; b. Conflicting results across studies.; c. Small sample sizes and unclear confidence intervals.; d. Some studies did not adequately adjust for confounders.; e. Relatively consistent results, with some differences observed.; f. Reasonable sample sizes. *Severity of periodontitis:* Based on data from Babür et al. [3], Agrawal et al. [4], Thomas [6] and Zhang et al. [8]. *Overall periodontal health status:* Based on data from Yakar et al. [5], Yakar et al. [7], Haas et al. [14], Agrawal et al. [15] and Scardina and Messina [16]

## Discussion

This systematic review is the first to synthesize current evidence on the clinical impact of menopause on periodontal health through a directly comparing postmenopausal and premenopausal women. By focusing on clinical parameters and disease progression, the findings reveal a tendency toward greater periodontal attachment loss, deeper probing depths, and a higher prevalence of advanced periodontitis in postmenopausal women. While these results are derived from observational studies, they suggest a potential role of hormonal changes in modulating periodontal disease progression. However, the certainty of this evidence, as assessed by the GRADE approach, remains moderate to low.

Declining estrogen levels during menopause have been associated with negative effects on periodontal health through multiple mechanisms [17]. Studies demonstrate increased bone metabolism due to estrogen deficiency [18]. Among the clinical periodontal parameters analyzed in this systematic review, CAL and PPD appeared to be the most consistently affected by menopausal status. Several studies included reported significantly higher mean values of CAL and PPD in postmenopausal women compared to premenopausal counterparts. While other parameters such PI, GI, and BOP showed variable results across studies, the consistent elevation in CAL and PPD highlights the clinical relevance of hormonal changes in periodontal status.

In addition to clinical findings, inflammatory biomarkers and tissue-level changes have been investigated as potential mediators of periodontal deterioration in postmenopausal women. Agrawal et al. [4] reported significantly higher salivary levels of TNF-α in postmenopausal women, a cytokine strongly associated with increased probing depth and clinical attachment loss. These findings support the hypothesis that estrogen deficiency enhances local inflammatory responses, amplifying periodontal breakdown [18].

Estrogen deficiency has been shown to exacerbate gingival inflammation and compromise epithelial barrier function in response to *Porphyromonas gingivalis* [19]. Experimental models indicate that 17β-estradiol attenuates the expression of pro-inflammatory cytokines, including IL-1β and IL-6, restores tight junction protein integrity, and mitigates alveolar bone loss following bacterial challenge. These findings underscore the mechanistic link between hormonal decline and periodontal tissue degradation in postmenopausal women [19]. In contrast, Babür et al. [3] found no significant differences in OPG levels in GCF, suggesting that bone remodeling via the RANKL/OPG may be more influenced by local disease severity than by menopausal status alone. Notably, Yakar et al. [5] observed that cytokine differences lost statistical significance after adjustment for age, pointing to inflammaging as a relevant systemic modifier. Together, these findings suggest that while menopause contributes to an enhanced pro-inflammatory and tissue-degenerative environment, its clinical impact is modulated by both systemic factors and local periodontal conditions.

Menopause is commonly diagnosed based on clinical symptoms rather than biochemical confirmation, and serum estrogen measurements are not used in dental settings. However, emerging evidence indicates that salivary estradiol may offer a practical, non-invasive biomarker to support the identification of menopausal status and inform periodontal treatment strategies in postmenopausal women [20]. Studies have shown that salivary estradiol levels are significantly lower in 95% of postmenopausal women compared to premenopausal controls. This decline correlates with symptoms such as xerostomia and increased salivary calcium, both factors associated with poorer periodontal outcomes [20].

The interpretation of the findings from this review must also consider the influence of key confounding factors that were inconsistently reported or inadequately controlled across the included studies. Our risk of bias analysis (Fig. 2) reveals that six of the nine studies had a critical or moderate risk of bias precisely due to such confounding. Specifically, fundamental confounders such as oral hygiene practices and socio-economic status were largely ignored. Variables such as age, diabetes mellitus, smoking, and diet are well-established modifiers of periodontal inflammation, yet these factors were not adequately adjusted for in several studies [4, 6–8, 15, 16]. Oral hygiene practices, socio-economic status, systemic conditions and smoking status were only accounted for in one study [14]. Dietary influences, though critical for microbial balance and immune modulation [21, 22], were not addressed in any study. Socio-economic status is a powerful determinant of health that influences diet, stress levels, health literacy, and access to regular dental care, all of which are independent risk factors for periodontitis [1]. Evidence shows that poor glycemic control and pro-inflammatory diets promote subgingival dysbiosis and enhance inflammatory signaling [23–25], while smoking alters host immunity and favors pathogenic colonization [22]. The cumulative omission of such variables limits the ability to isolate the effect of menopause and likely contributed to the heterogeneity observed, reinforcing the moderate to low certainty of evidence in this review.

A significant limitation impacting the internal validity of this review's findings is the heterogeneity in the methodology used to confirm menopausal status across the included studies. As detailed in Table 1, methods ranged from biochemical confirmation via serum estradiol levels [7] to clinical criteria based on self-reported amenorrhea [14] or were not reported at all. This inconsistency introduces a high risk of misclassification bias, where women in the perimenopausal transition could have been incorrectly classified as either pre- or postmenopausal. Such misclassification could dilute the true effect of menopause on periodontal outcomes, potentially underestimating the association and contributing to the inconsistent results observed across studies.

A major challenge in synthesizing the evidence was the considerable heterogeneity in the assessment and reporting of periodontal outcomes. As shown in Table 1, the included studies used disparate methods to define periodontal disease. For instance, Yakar et al. [7] utilized the 2017 World Workshop classification to define Stage 3–4 periodontitis, whereas Agrawal et al. [15] and others relied on continuous variables such as mean PPD and CAL. Still others, like Thomas [6], used broad, non-standardized categories like chronic periodontitis. This lack of a uniform case definition is a well-documented challenge in periodontal research that severely limits the ability to compare disease prevalence and severity across populations. Consequently, this methodological inconsistency prevented a quantitative synthesis of the data via meta-analysis and contributed significantly to the low-to-moderate certainty of the evidence presented in this review.

A critical methodological flaw across the body of evidence is the inconsistent and largely inadequate handling of the duration since menopause. The physiological consequences of estrogen deficiency, particularly on bone metabolism, are not static but cumulative over time; bone loss, for instance, is known to accelerate in the first few years following menopause before stabilizing at a slower rate [2]. Six of the nine included studies failed to report or adjust for this key variable. While there were exceptions, Zhang et al. [8] statistically adjusted for years since menopause and Agrawal et al. [4] controlled for it by limiting their sample to women within five years of menopause. This inconsistent approach prevents a clear analysis of a potential dose–response relationship, where time acts as the dose of estrogen deprivation, an oversight that obscures the true magnitude of the long-term impact of hormonal changes on periodontal health and represents a significant gap in the current literature.

Considering the biological plausibility and the clinical trends identified, menopause should be regarded as a relevant systemic modifier in the context of periodontal care [26]. The heightened severity of clinical attachment loss and probing depth observed in postmenopausal women warrants closer clinical monitoring and individualized prevention strategies. Periodontal risk assessment protocols should incorporate menopausal status alongside other established risk factors such as diabetes, smoking, and inflammatory dietary patterns. In this context, tailored approaches may help to mitigate disease progression and preserve periodontal stability. Integration between dental and medical teams is essential to address hormonal and metabolic influences in a comprehensive and patient-centered manner.

Hormonal shifts during menopause may critically influence the progression of periodontal disease [27, 28]. However, establishing causality remains challenging due to the predominance of observational designs and variability across studies. Specifically, the cross-sectional nature of eight of the nine included studies prevents the establishment of a clear temporal relationship between the onset of menopause and the progression of periodontal disease. Without this temporal sequence, demonstrating that the menopause occurred before worsened periodontal parameters, it is impossible to infer a cause-and-effect relationship. Therefore, the findings must be interpreted as associations rather than direct causal evidence, highlighting a critical area for future longitudinal research. While previous reviews have addressed this association [29–31], none have incorporated recent data using the GRADE and ROBINS-I frameworks, nor explicitly excluded hormone therapy as a confounder. In this context, the methodological approach of the present review offers added value. By excluding hormone therapy and stratifying outcomes by disease status, this review provides a more focused assessment of the direct impact of menopause on periodontal health. The application of ROBINS-I and the GRADE framework [13], both rarely used in this area, enabled a transparent evaluation of bias and evidence of certainty, reinforcing the relevance of menopause as a potential systemic modifier.

To overcome the limitations of the current literature, future research must adopt a more rigorous and standardized approach. Instead of merely calling for high-quality prospective studies, we prescribe a clear methodological framework. Firstly, longitudinal cohort studies are essential to establish a temporal relationship between menopause and periodontal disease progression, allowing for causal inference. Secondly, menopausal status must be defined using standardized, objective criteria, such as the Stages of Reproductive Aging Workshop + 10 (STRAW + 10) guidelines, which incorporate both menstrual history and endocrine markers, thereby minimizing misclassification bias [32]. Thirdly, periodontal outcomes must be assessed using full-mouth examinations and defined according to the globally accepted 2017 World Workshop classification, with mandatory reporting of examiner calibration to ensure data reliability and facilitate future meta-analyses [33]. Finally, studies must rigorously control for all key confounders, including, critically, the duration since menopause. Moreover, incorporating the analysis of local biomarkers from gingival crevicular fluid or saliva could further elucidate the underlying biological mechanisms [34]. Adopting this comprehensive framework is essential for generating high-certainty evidence to inform clinical practice and improve the periodontal health of postmenopausal women.

## Conclusion

Menopause appears to negatively impact periodontal health, with postmenopausal women showing greater clinical attachment loss, deeper probing depths, and increased signs of inflammation. While current evidence points toward this association, the overall certainty remains moderate to low due to methodological limitations and variability across studies. Well-designed longitudinal studies are needed to confirm these findings and to support the development of personalized periodontal care strategies for postmenopausal women.

## Supplementary Information

Below is the link to the electronic supplementary material.

Supplementary file1 (PDF 68 KB)

## Data Availability

Data sharing is not applicable to this article as no new data were created or analyzed in this study.
